# 1.C. Workshop: What has the EU ever done for my health system? Using EU tools effectively for health system change

**DOI:** 10.1093/eurpub/ckac129.009

**Published:** 2022-10-25

**Authors:** Organised by: European Observatory on Health Systems and Policies, Chair persons: Dimitra Panteli (Belgium), Nick Fahy (UK)

## Abstract

Health systems face a broad range of evolving challenges: from infectious cross-border threats to climate change, potential backlogs caused by the pandemic and the concurrent burden of non-communicable diseases growing worldwide. They need to be dynamic to respond in ways that help them achieve their goals and adapt to an ever-changing landscape of health system stressors. While improving health and care systems is primarily the responsibility of Member States within the European Union (EU), the EU has a wide range of instruments that can potentially provide support. However, they are scattered across different policy areas and, for most of them, health systems strengthening is not among the principal objectives. This can make it challenging for Member States to identify, combine and make best use of the different support options in an effective way. The objective of this workshop will be to provide an overview of the different types of available EU instruments, while also delineating a range of practical examples of how countries have used them to address health system challenges and implement large-scale change. The European Observatory on Health Systems and Policies will kick off the workshop with a keynote presentation of its policy brief “EU support for improving health and care systems”, conceived in cooperation with the 2021 Slovenian Presidency of the EU Council as a toolbox for policy makers trying to navigate and maximise the potential of EU support tools. Beyond having to identify the best possible tools for health system transformation, policy makers must pave the way for system change and propel the implementation of innovative solutions at national, regional, and local levels. The new European Partnership on “Transforming Health and Care Systems” under Horizon Europe will join the workshop to discuss how to best support policy makers in bringing about organisational change and ensure European populations get access to innovative, sustainable, and high-quality health care. In addition, the workshop will give voice to insights from other relevant stakeholders, including a country-level perspective from Slovenia, an EU Member State which has successfully leveraged EU support to strengthen its health system in the past. This viewpoint will be complemented by an outlook from the European Commission into current priorities and upcoming opportunities under the most relevant EU support instruments for health systems, including EU4Health and Horizon Europe. Finally, the presenters and panellists will engage in an interactive discussion with the audience to help participants consider the possibilities and challenges of using EU tools for health system strengthening and to support them in designing their own initiatives.

**Key messages:**

• Reaping maximal benefits of EU support requires combining various instruments.

• Health system transformation is a context-specific process, yet it can greatly benefit from cross-country learning.

